# Energy Balance of a Typical U.S. Diet 

**DOI:** 10.3390/foods2020132

**Published:** 2013-03-28

**Authors:** Athanasios Alexandrou, Klaus Tenbergen, Diganta Adhikari

**Affiliations:** Jordan College of Agricultural Sciences and Technology, California State University-Fresno, Fresno, CA 93710, USA; E-Mails: ktenbergen@csufresno.edu (K.T.); diganta@csufresno.edu (D.A.)

**Keywords:** U.S. diet, energy sequestered, energy efficiency, energy balance

## Abstract

Today’s agriculture provides an ever increasing population with sufficient quantities of food. During food production, processing, handling and transportation, an amount of energy is invested into the various products. An energy analysis of a typical American diet provides policy makers, farmers and the public with the necessary information to evaluate and make informed decisions as to how to improve the efficient use of energy. At the same time, an informed consumer may become energy conscious and be able to make dietary choices based on food energy balance. This paper studies the energy sequestered in a typical American diet as defined in Food and Agriculture Organization of the United Nations, Statistics Division (FAOSTAT). The amount of energy incorporated in this diet of 3628 kcal (15.18 MJ) per person and day to produce, transport, handle and process the foods is calculated and found to have approximately 39.92 GJ (9.54 Gcal) sequestered per person and year. It is shown that a diet in line with the United States Department of Agriculture (USDA) recommendation of around 2100 kcal (8.79 MJ) per day person will result in a reduction of energy inputs by 42% on an annual basis. This reduction for the whole population of the United States of America (USA), corresponds to approximately 879 million barrels of oil equivalent (boe) savings. Energy efficiency for the food categories studied varies from 3.4% to 56.5% with an average of 21.7%. Food energy efficiency can be further improved in some food categories through either a reduction of energy inputs or yield increase.

## 1. Introduction

Energy is undoubtedly related to human progress and the technological achievements of our civilization. The major sources of energy for human beings have changed over time. It took centuries for our society to pass from the wood, animal and human energy based economy of the past to the fossil fuel energy based economy of today. Agriculture mirrors the changes of our society. At its initial stage, agriculture was based in human and animal power. Currently, agriculture in the United States is a power intensive industry where extensive amounts of energy, either direct or indirect, are used to secure a maximum yield, and if population projections hold truth, the agricultural sector of the economy may be asked to increase its productivity to provide sufficient nutrients for a continuously increasing population. 

An energy analysis of various food products incorporated into a typical American diet provides policy makers, farmers and public with the necessary information to evaluate and make informed decisions as to how to improve the efficient use of energy. At the same time, an informed consumer may become energy conscious and be able to make dietary changes based wholly or partially on energy balance information. The last few decades and specifically after the oil crisis, public, researchers and policy makers showed an interest in energy issues. There is an increasing body of published work related to energy use in agriculture [1] and to the energy use in food systems [2]. However, little attention has been paid to the energy sequestered into edible food and its energy efficiency. The first step toward identifying energy efficiency of a diet is to identify the type of foods consumed and the per capita food supply needs of the diet and then to estimate the energy balance of the system. 

USDA recommends foods for a typical diet, but these foods and caloric values do not necessarily represent the actual U.S. consumption. In this paper, the per capita food supply for the U.S. was obtained from FAOSTAT, Food Balance Sheets [3]. FAOSTAT [4] states that: “a food balance sheet presents a comprehensive picture of the pattern of a country’s food supply during a specified reference period”. The latest data available for the U.S.A. is for the year 2009 [3], which shows 15.4 MJ (3688 kcal) caloric consumption per person and day. From this caloric consumption, 72.5% (11.2 MJ or 2675 kcal) is based on vegetal products and 27.5% (4.2 MJ or 1013 kcal) is based on animal products.

The energy input of a typical diet of an American citizen is studied in this paper. The energy output is also analyzed and food energy balance and food efficiency are derived. The units used in this paper are widely used to describe the physical quantity and may not necessarily be units of the international system (SI). The authors chose to use these units to facilitate the readers who may not necessarily be familiar with the corresponding international system unit. 

## 2. Materials and Methods

The energy analysis conducted in this study quantifies the energy inputs required to produce a specific agricultural product which is part of the typical American diet. FAOSTAT data represent mostly commercialized major food crops. Non-commercial production (*i.e.*, home produce) is not included [4]. The accuracy of the data is one of the issues of Food Agriculture Organization (FAO), Food Balance Sheets, since accurate or reliable production statistics may not be available for all commodities. Furthermore, the accuracy of the balance sheet depends on the reliability of the statistical data on population, supply and utilization of food and their nutritive value and waste [4] it. In the FAO, Food Balance Sheets handbook [4] is stated that “… the food balance sheets represent only the average supply available for the population and do not necessarily indicate what is actually consumed by individuals”. 

Data used in this study do not include pre-harvest losses or losses in the field that are common during various stages of the crop production process. Moreover, food available in the food balance sheet, reflect only the quantities reaching the consumer [4]. The amount of food actually consumed may be lower depending on the degree of losses of edible food [4]. The energy analyses used in this study are based on yield which is defined as the crop production removed from the field, and is expressed as quantity harvested per unit of area. 

Post-harvest food waste also referred to as food losses or spoilage is another source of discarded food. Food loss refers to the decrease in food quality or quantity, which makes it unfit for human consumption [5]. Kader [6] estimated that approximately one third of all fresh fruit and vegetable produced worldwide is lost before it reaches consumers. He estimated that food losses in the U.S. range from 2 to 23 percent depending on the commodity with an overall average of 12 percent. For the purposes of this study, all energy inputs were considered in the analyses presented, independent if some of the food was eventually wasted. 

In order to derive the energy sequestered in various food types, first the energy input used for the production of the food was calculated. Then the energy required for transportation, processing and handling was assessed. The sum of the two components constitutes the energy sequestered into a food. 

In relation to the energy sequestered in a product, it is assumed that energy passed or dissipated by a process is passed on in the products of that process [7]. For example the embodied energy in a cantaloupe at the farm gate, includes all the energy used in the production of this cantaloupe, direct and indirect. Leaving the farm, the fresh produce is transported for example, from the fields of Central Valley of California to the supermarket in Los Angeles, in refrigerated containers. An amount of energy is used to transport and keep refrigerated the produce. When the produce is used as food, this amount of energy is considered to be sequestered in the produce. The work from various authors was used to estimate energy inputs from crop agricultural operations. Data were obtained from studies carried out in the U.S. and in other countries at different time periods. For the purposes of this study is assumed that the results of the energy analyses used in this study are valid today. 

### 2.1. Energy Inputs

#### 2.1.1. Energy Input for Crop Production

Most work in the area of energy analysis for food systems was initiated after the oil crisis by David Pimentel [1]. It should be noted that although the analytical methods employed for energy analysis of various agricultural products are well established and documented, there are still inconsistencies regarding the methodology and various assumptions used. This poses a problem since some of the energy analysis results may not be compatible with similar work done by other researchers. In this study, the results of energy analyses from various authors have been used and any inconsistencies regarding methodology and assumptions used were considered inconsequential. 

The energy required for agricultural production is divided into direct and indirect categories [8]. Direct energy inputs included those quantities that were consumed during actual production operations. For example the amount of energy contained in the fuel used. Indirect energy inputs were those required to manufacture and maintain durable goods, such as tractors and other equipment and machinery, as well as other materials used for agricultural production [9,10]. The result of this analysis is the amount of energy required to produce one unit of weight of the agricultural product. 

It must be noted that the analytical methods used in most agricultural energy analysis studies, do not take into account solar energy. Solar energy either as radiation or heat participates into the agricultural production in a rather major manner. It is mostly considered a “free” subsidy in the energetic analysis of agricultural systems [11]. In this study solar energy was not considered into the calculations.

#### 2.1.2. Energy Input for Livestock Production

Livestock production is a poor converter of energy because of the inherent inefficiencies of the energy transformation of green plants into biomass. Moreover, a major share of the energy intake of livestock is spent on keeping up the metabolism and consequently there are higher energy demands of creating fat and protein. Table 1 shows the energy input (direct and indirect) per unit of animal food product [12]. For the purposes of this study these values of energy inputs were used to estimate the energy input for livestock production.

**Table 1:** Direct and indirect energy inputs into the animal food product [12].

Food product	Animal feed conversion	Direct and indirect energy inputs	Average energy input
Chicken	4.2 kg/kg edible meat	25–35 MJ/kg meat	30 MJ/kg (7170 kcal/kg)
Pork	10.7 kg/kg edible meat	25–70 MJ/kg meat	47.5 MJ/kg (11,353 kcal/kg)
Beef (feedlots)	31.7 kg/kg edible meat	80–100 MJ/kg meat	90 MJ/kg (21,511 kcal/kg)
Laying hens	4.2 kg/kg eggs	450–500 MJ/year	475 MJ/kg (113,528 kcal/kg)
Dairy milk	0.7 kg/liter milk	5–7 MJ/liter of fresh milk	6 MJ/liter (1434 kcal/kg)

#### 2.1.3. Energy Input for Fish Production

As with any food production system, fisheries require an amount of energy input in the form of capital equipment, labor and fuel to obtain the desired output [13]. Contrary to other sectors of agriculture production, direct energy inputs to fisheries constitute the largest fraction of energy use (75%–90%) [13]. 

Bardach [14] estimated the fish production per liter of fuel for three different fishing technologies and concluded that the average fish catch was 7.9 kg. In this study, it was assumed that this was the average catch per liter of fuel and that the diesel fuel contains 35.9 MJ (8580 kcal) per liter. He recommended the increase of the amount of diesel fuel by 17.5% to compensate for indirect energy. The above mentioned assumptions were adopted and reflected in Table 2.

**Table 2:** Food supply and energy input for a typical American diet for the year 2004 [15,16].

Item (1)	Food supply quantity kg/capita/year (%) (2)	Food supply kcal/capita/day (MJ/capita/day) (3)	Energy input for agricultural production, kcal/kg (MJ/kg) (4)	Energy input for Transportation, Handling and Processing, kcal/kg (MJ/kg) (5)	Average Input Energy, kcal/kg (MJ/kg) (6)	Annual Energy Input kcal/capita/year (MJ/capita/year) (7)	Percentage of energy input per food category, % (8)
Grains ^1^	111	827	1716	6794	8510	907,757	9.52
	(12.03%)	(3.46)	(7.18)	(28.43)	(35.61)	(3798.05)	
Vegetables ^2^	126	193	766	6790	7556	1,515,979	15.90
	(13.65%)	(0.81)	(3.20)	(28.41)	(31.61)	(6342.86)	
Sugar & Sweeteners ^3^	71	663	5924	6797	12,721	899,404	9.43
	(7.69%)	(2.77)	(24.79)	(28.44)	(53.22)	(3763.11)	
Pulses ^4^	4	40	1937	6652	8588	36,804	0.39
	(0.43%)	(0.17)	(8.10)	(27.83)	(35.93)	(153.99)	
Treenuts & peanuts ^5^	4	65	2940	6827	9767	41,999	0.44
	(0.43%)	(0.27)	(12.30)	(28.56)	(40.87)	(175.72)	
Fats and Oils ^6^	33	778	1509	6823	8332	271,232	2.84
	(3.58%)	(3.26)	(6.31)	(28.55)	(34.86)	(1134.83)	
Fruits (Excluding Wine) ^7^	124	142	674	6789	7464	923,428	9.68
	(13.43%)	(0.59)	(2.82)	(28.41)	(31.23)	(3863.62)	
Meat & Fish ^8^	176	532	13,345	6805	17,947	2,686,313	28.17
	(19.07%)	(2.23)	(55.84)	(28.47)	(75.09)	(11,239.53)	
Eggs ^9^	15	57	1469	6762	8231	122,636	1.29
	(1.63%)	(0.24)	(6.15)	(28.29)	(34.44)	(513.11)	
Dairy ^10^	259	382	1434	6812	8246	2,131,590	22.35
	(28.06%)	(1.60)	(6.00)	(28.50)	(34.50)	(8918.57)	
*Total*	*923*	*3679*	*31,714*	*67,851*	*97,363*	*9,537,142*	*100*
	*(100)*	*(15.39)*	*(132.69)*	*(283.89)*	*(407.37)*	*(39,903.40)*	

^1^ Grains include: wheat, rice barley, maize, rye, oats, sorghum; ^2^ Vegetables include: cassava, soybeans, potatoes, sweet potatoes, yams, roots, olives, tomatoes, onions, pimiento; ^3^ Caloric sweeteners include: sugar, sugar beet, sugar cane, honey; ^4^ Dry beans, peas, and lentils include: beans, peas; ^5^ Tree nuts and peanuts include: groundnuts, sunflower seed, rape and mustard seed, coconut, sesame seed; ^6^ Fats and oils include: soybean oil, groundnut oil, sunflower seed oil, rape and mustard oil, cottonseed oil, palm kernel oil, coconut oil, olive oil, maize germ oil, oilcrops oil, butter, raw animal fats; ^7^ Fruits include: oranges, mandarins, lemons, limes, grapefruit, citrus, bananas, plantains, apples, pineapples, dates, grapes, coffee, cocoa beans; ^8^ Meat & fish include: bovine meat, mutton and goat meat, pig meat, poultry meat, freshwater fish, demersal fish, pelagic fish, crustaceans, cephalopods, molluscus; ^9^ Eggs include: eggs; ^10^ Dairy include: milk, excluding butter.

#### 2.1.4. Energy Input for Transportation, Processing and Handling

Cuellar and Webber [15] calculated the amount of energy used to produce food from agriculture including energy used for transportation, processing and handling for the year 2004 and for ten different food categories using the mass-based relative intensity values. The energy required for transportation, processing and handling was calculated in trillion of British thermal units (Btus) per food category. This energy was converted in kcal per kg of food and used in this study. 

The use of data from the year 2004 in the Cuellar and Webber [15] study obliged the authors to use FAOSTAT, Food Balance Sheets data for the same year [16] to avoid any uncertainties that may be created by using data sets from different years. For the year 2004, an American consumed 3853 kcal (16.12 MJ) per day. From this caloric consumption, 72.8% (2805 kcal or 11.74 MJ) is based on vegetal products and 27.2% (1047 kcal or 4.38 MJ) is based on animal products. It must be noted that the difference between the 3853 kcal (16.12 MJ) per person per day and the USDA recommended ~2100 kcal (8.79 MJ) per person per day may be due to overeating and food discarded after being fully processed and distributed [17]. 

The total amount of energy required for transportation, handling and processing for each food category was determined by using data from [15] and dividing by the mass of food.

Table 3 shows the data used for calculation of a typical American diet energy input for grains using FAOSTAT data for the year 2004 [16] and data from Cuellar and Webber [15] for energy for transportation, handling and processing. The second column shows data on food supply quantity per person and year. The third column includes data on energy input for crop production in kcal/kg (MJ/kg) while the total energy input per kilogram and per capita and year was also calculated. The total energy input for the other food categories is been calculated similarly.

**Table 3:** Grain food supply for the year 2004 [15,16].

Item(1)	Food supply quantity, kg/capita/year (2)	Energy input for agriculture, kcal/kg, (MJ/kg) (3)	Energy input for transportation, handling and processing, kcal/kg (MJ/kg) (4)	Total input energy, kcal/kg (MJ/kg) (5) = (3) + (4)	Annual energy input kcal/capita/year (MJ/capita/year) (6) = (2) × (5)	References (7)
Wheat	83.4	1236	6794	8030	669,690	[18]
		(5.17)	(28.43)	(33.60)	(2801.98)	
Rice	8.2	2645	6794	9439	77,399	[19]
		(11.01)	(28.43)	(39.49)	(323.84)	
Barley	0.5	815	6794	7609	3804	[20]
		(3.41)	(28.43)	(31.84)	(15.91)	
Maize	13.2	1889	6794	8683	114,614	[21]
		(7.90)	(28.43)	(36.33)	(479.54)	
Rye	0.3	1216	6794	8010	2403	[22]
		(5.09)	(28.43)	(33.51)	(10.05)	
Oats	3.7	742	6794	7536	27,883	[23]
		(3.10)	(28.43)	(31.53)	(116.66)	
Sorghum	1	3469	6794	10,263	10,263	[24]
		(14.51)	(28.43)	(42.94)	(42.94)	
Cereals, Other (average of the above)	0.2	1716	6794	8510	1702	
		(7.18)	(28.43)	(35.61)	(7.12)	
*Total*	*110.5*				*907,757 (3798.05)*	

### 2.2. Energy Output

The energy contained in edible food is called output energy or available energy, and can be defined as the amount of energy present in food after digestive and urinary losses are deducted from gross energy [25]. This energy is in the form of carbohydrate, fat and protein minus the amount present in feces [26]. It should be noted that even for healthy humans, the same diet may result in different amounts of available energy to different people [26]. This energy is what is used by the USDA to determine the energy available in a food and is used in this study. Data for the caloric value of the American diet were obtained from FAOSTAT [16].

## 3. Results and Discussion

A typical American diet contains the ten food categories (Table 2) as defined by Cuellar and Webber [15]. The food types included in each category have been identified and listed in Table 2. Columns (4) and (5) of Table 2, show the amount of energy input for production of one kilogram of the food and the energy input for transportation, handling and processing per kilogram of food. It can be seen that for some products the amount of energy input for transportation, handling and processing per unit of weight, exceeds the energy input for production. For the meat and fish food category the energy input for production is the largest for all categories listed. It should be noted that tea (leave), pepper (spice), wine, beer, and alcoholic beverages were not categorized and consequently were not listed in Table 2. This happened because of the inexistence of data concerning energy inputs during the production of the above mentioned foods. Consequently, the total caloric value of the diet examined is being reduced to 3679 kcal (15.39 MJ), which corresponds to the amount of calories of the above mentioned foods minus the caloric value of foods were data of energy input during their production were not available. 

There is less confidence in the results of the analysis for some food categories compared to others. The fats and oils food category results for example, are based on a smaller number of foods than other categories. The authors were able to find references for only two out of twenty three items included in this category related to the energy input for production. The average of the dataset was eventually used to derive the energy input for agricultural production for the foods were real data were not available. This undermined the validity of the data specifically when referred to energy efficiency. 

Table 2 also shows that the average American diet (without including alcohol) requires an annual energy input per person of approximately 39.92 GJ (9.54 Gcal) to produce, transport, handle and process the foods. From the ten food categories listed, meat and fish, milk and vegetables have sequestered the most energy and contribute to over 66% of the total energy sequestered in the annual American diet. The meat and fish category with 28.17%, milk with 22.35% and vegetables with 15.90% of the annual energy input per person. By weight the same categories total almost 61% of the total annual consumption per person. In detail, meat and fish with 19.07%, milk with 28.06% and vegetables with 13.6% of the annual weight consumption per person.

Table 4 shows the energy input on an American diet in line with the USDA recommendation of around 2100 kcal (8.79 MJ) per day and person. The daily food supply of Table 2 was reduced by approximately 42% to obtain the 2100 kcal (8.79 MJ) daily food intake recommended by USDA. Consequently, Table 4 assumes that the diet contains the same food quantities and types of food with the ones listed in FAOSTAT [16]. All food categories have been reduced by the same percentage. It can be seen that a reduction of the intake calories by approximately 42%, from 3628 kcal (15.18 MJ) per person and day to around 2100 kcal (8.79 MJ), results in a similar percentage reduction of the energy input required to produce, transport, handle and process the food.

**Table 4:** Food supply and energy input for a typical American diet (2100 kcal or 8.79 MJ).

Item	Food supply quantity kg/capita/year	Food supply kcal/capita/day (MJ/capita/day)	Annual Energy Input kcal/capita/year (MJ/capita/year)
Grains	63	471	517,421
		(1.97)	(2164.89)
Vegetables	72	110	864,108
		(0.46)	(3615.43)
Sugar & Sweeteners	40	378	512,660
		(1.58)	(2144.97)
Pulses	2	23	20,978
		(0.10)	(87.77)
Treenuts & peanuts	2	37	23,939
		(0.15)	(100.16)
Fats and Oils	19	443	154,602
		(1.85)	(646.85)
Fruits (Excluding Wine)	71	81	526,354
		(0.34)	(2202.27)
Meat & Fish	100	303	1,531,198
		(1.27)	(6406.53)
Eggs	9	32	69,903
		(0.13)	(292.47)
Dairy	148	218	1,215,006
		(0.91)	(5083.59)
*Total*	*526*	*2097*	*5,436,171*
		*(8.77)*	*(22,744.94)*

In real terms, the reduction is around 4.1 Gcal per person and year. In terms of energy this amount corresponds to 2.93 barrels of oil equivalent (boe) (1 boe = 1,400,000 kcal = 5186.52 MJ). This amount is rather significant and when it is multiplied by the population of the USA which is around 300 million, it provides the potential for energy reduction of 879 million boe per year or provides for a capacity to feed more people. 

Table 5 shows the net caloric value and energy efficiency of the foods involved in an American diet as defined in FAOSTAT [16]. It can be seen that energy efficiency ranges between 3.4% for vegetables to almost 57% for treenuts and peanuts with an average of 21.7%. There is some room for further improvement in some food categories through either a reduction of energy inputs or yield increase. It should be noted that in this study energy input from solar energy was not considered.

**Table 5:** Food supply and energy efficiency for a typical American diet for the year 2004 [15,16].

Item (1)	Food supply quantity kg/capita/year (2)	Food supply kcal/capita/day (MJ/capita/day) (3)	Annual Energy Input kcal/capita/year (MJ/capita/year) (5)	Net Caloric value, kcal/year (MJ/year) (6)	Energy efficiency, % (7) = (6)/(5)
Grains	111	827	907,757	301,855	33.25
		(3.46)	(3798.06)	(1262.96)	
Vegetables	126	193	1,515,979	51,830	3.42
		(0.81)	(6342.86)	(216.86)	
Sugar & Sweeteners	71	663	899,404	241,995	26.91
		(2.77)	(3763.11)	(1012.51)	
Pulses	4	40	36,804	14,600	39.67
		(0.17)	(153.99)	(61.09)	
Treenuts & peanuts	4	65	41,999	23,725	56.49
		(0.27)	(175.72)	(99.27)	
Fats and Oils	33	778	271,232	283,970	N/A ^1^
		(3.26)	(1134.83)	(1188.13)	
Fruits	124	142	923,428	51,830	5.61
		(0.59)	(3863.62)	(216.86)	
Meat & Fish	176	532	2,686,313	181,770	6.77
		(2.23)	(11,239.53)	(760.53)	
Eggs	15	57	122,636	20,805	16.96
		(0.24)	(513.11)	(87.05)	
Dairy	259	382	2,131,590	139,430	6.54
		(1.60)	(8918.57)	(583.38)	
*Total*	*923*	*3628*	*9,537,142*	*1,311,810*	*21.7 ^2^*
		*(15.18)*	*(39,903.49)*	*(5488.61)*	

^1^ There is no confidence in the data used for this food category to derive the energy efficiency; ^2^ The average does not include the fats and oils category.

## 4. Conclusions

Energy sequestered in a typical American diet as defined in FAOSTAT [16] was estimated in this study. The amount of energy incorporated in this diet of 3628 kcal (15.18 MJ) per person per day to produce, transport, handle and process the foods is calculated to reach approximately 39.92 GJ (9.54 Gcal) per person and year. Meat and fish, milk and vegetables are the food categories that contribute to over 66% of the total energy sequestered in the annual American diet. It is shown that a diet in line with the USDA recommendation of around 2100 kcal (8.79 MJ) per day and person will result in a reduction of energy inputs by 42% on an annual basis. On an annual basis and for the whole population of the U.S.A., this reduction corresponds to approximately 879 million boe savings. Energy efficiency for the food categories studied varies from 3.4% to 56.5 with an average of 21.7%. There is some room for further improvement in energy efficiency for some food categories through either a reduction of energy inputs or yield increase. 
